# Chemical Problems

**Published:** 1885-10

**Authors:** 


*Chemical Problems.*—By Dr. Karl Stammer. Translated from the second German edition, with explanations and answers, by W. S. Hoskinson, A. M., of Wittenberg College, Ohio. Publishers: P. Blackiston, Son & Co., Philadelphia. 1885. Price, 75 cents.

The text is in the form of questions to which answers are given at the end of the volume, which comprises one hundred and nine pages. Part First relates to the recognized elements, and Part Second to approximate ratios, temperature, atmospheric pressure and mixed problems, making a compact and useful text-book for the study of chemical problems.

